# Supplementary material for: A multistate assessment of population normalization factors for wastewater-based epidemiology of COVID-19
Source: PLoS One. 2023 Apr 12;18(4):e0284370. doi: 10.1371/journal.pone.0284370 (PMC10096268; doi:10.1371/journal.pone.0284370)
Supplement: S1 Table — (DOCX) [file pone.0284370.s001.docx]

**S1 Table. Influent wastewater processing methods used by each study site concentration, extraction, and molecular analysis of SARS-CoV-2 RNA.**

| **Study Site** | **Concentration Method** | **Extraction Method** | **Molecular Analysis** | |
| --- | --- | --- | --- | --- |
|  |  |  | RT-ddPCR | rRT-qPCR |
| North Carolina | Membrane filtration with acidification and MgCl_2_ | Nuclisens magnetic bead extraction kit | + | - |
| Wisconsin | Membrane filtration with MgCl_2_ | Promega ht TNA kit &  Qiagen RNeasy Power Microbiome Kit | + | + |
| Colorado | Innovaprep ultrafiltration | Qiagen QIAamp buffers with epoch columns | + | - |
| Virginia | Membrane filtration with acidification and MgCl_2_ & Innovaprep ultrafiltration | Nuclisens magnetic bead extraction kit | + | - |
| California | Membrane filtration with acidification and MgCl_2_ | 4S method, Nuclisens magnetic bead extraction kit, quick RNA fecal/soil microbe microprep kit, Zymo environmental water RNA kit | + | + |
| Ohio | Concentrating pipette ultrafiltration, Membrane filtration with/without sample acidification, Ultrafiltration 30kda centricon unit | Qiagen QIAamp viral RNA,  Qiagen allprep powerviral DNA/RNA kit, Qiagen power water kit, Promega wastewater large volume TNA capture kit, Trizol-chloroform extraction | + | + |
