# Supplementary material for: A multistate assessment of population normalization factors for wastewater-based epidemiology of COVID-19
Source: PLoS One. 2023 Apr 12;18(4):e0284370. doi: 10.1371/journal.pone.0284370 (PMC10096268; doi:10.1371/journal.pone.0284370)
Supplement: S2 Table — (DOCX) [file pone.0284370.s002.docx]

**S2 Table. Spearman correlation coefficient analysis of the fecal indicator raw wastewater concentration (Log_10_GC/L), fecal indicator flow normalized wastewater concentration (Log_10_GC/Day), and flow rate (L/Day) with the estimated population served across each study site. NR = Not Reported.**

| **Study Site** | **Fecal Indicator**  **Raw Concentration Correlation Coefficient**  **(P-Value)** | | | **Fecal Indicator**  **Flow Normalized Concentration Correlation Coefficient**  **(P-Value)** | | | **Flow Rate Correlation Coefficient**  **(P-Value)** |
| --- | --- | --- | --- | --- | --- | --- | --- |
|  | **PMMoV** | **F+ Coliphage** | **crAssphage** | **PMMoV** | **F+ Coliphage** | **crAssphage** |  |
| North Carolina | 0.31 (<0.001) | NR | NR | 0.83 (<0.001) | NR | NR | 0.92 (<0.001) |
| Wisconsin | 0.30 (<0.001) | NR | NR | 0.89 (<0.001) | NR | NR | 0.96 (<0.001) |
| Colorado | 0.27 (<0.001) | 0.08 (<0.001) | NR | 0.82 (<0.001) | 0.80 (<0.001) | NR | 0.88 (<0.001) |
| Virginia | -0.04 (0.47) | NR | NR | 0.29 (<0.001) | NR | NR | 0.76 (<0.001) |
| California | 0.56 (<0.001) | NR | NR | 0.81 (<0.001) | NR | NR | 0.94 (<0.001) |
| Ohio | 0.03 (0.41) | NR | 0.17 (<0.001) | 0.55 (<0.001) | NR | 0.62 (<0.001) | 0.91 (<0.001) |
