# Supplementary material for: A multistate assessment of population normalization factors for wastewater-based epidemiology of COVID-19
Source: PLoS One. 2023 Apr 12;18(4):e0284370. doi: 10.1371/journal.pone.0284370 (PMC10096268; doi:10.1371/journal.pone.0284370)
Supplement: S3 Table — (DOCX) [file pone.0284370.s003.docx]

**S3 Table. Spearman correlation coefficients (P-Value) of each normalization category for the SARS-CoV-2 wastewater concentrations and the sewershed-level COVID-19 cases for each study site. NR = Not Reported.**

| **Genetic Target (Normalization Category)** | **North Carolina** | **Wisconsin** | | | **Colorado** | **Virginia** | **California** | | | **Ohio** | | |
| --- | --- | --- | --- | --- | --- | --- | --- | --- | --- | --- | --- | --- |
|  |  | Overall | RT-ddPCR | rRT-qPCR |  |  | Overall | RT-ddPCR | rRT-qPCR | Overall | RT-ddPCR | rRT-qPCR |
| CDC N1  (Raw) | 0.54 (<0.001) | 0.04 (0.006) | 0.45 (<0.001) | 0.20 (<0.001) | 0.53  (<0.001) | 0.36 (<0.001) | 0.56 (<0.001) | 0.83 (<0.001) | 0.63 (<0.001) | 0.04 (0.189) | NR | 0.04 (0.189) |
| CDC N2  (Raw) | 0.59 (<0.001) | -0.01 (0.37) | 0.42 (<0.001) | 0.15 (<0.001) | NR | 0.62 (<0.001) | 0.82 (<0.001) | 0.82 (<0.001) | 0.91 (<0.001) | 0.19 (<0.001) | 0.48 (<0.001) | 0.12 (<0.001) |
| CDC N1  (Flow) | 0.33 (<0.001) | 0.55 (<0.001) | 0.72  (<0.001) | 0.58 (<0.001) | 0.84  (<0.001) | 0.47 (<0.001) | 0.79 (<0.001) | 0.82 (<0.001) | 0.79 (<0.001) | 0.13 (<0.001) | NR | 0.13 (<0.001) |
| CDC N2  (Flow) | 0.33 (<0.001) | 0.47 (<0.001) | 0.69  (<0.001) | 0.52  (<0.001) | NR | 0.70 (<0.001) | 0.84 (<0.001) | 0.84 (<0.001) | 0.90 (<0.001) | 0.50 (<0.001) | 0.73 (<0.001) | 0.39 (<0.001) |
| CDC N1  (Fecal) | 0.52 (<0.001) | -0.02 (0.09) | 0.44 (<0.001) | 0.13 (<0.001) | 0.63  (<0.001)^a^  0.50  (<0.001)^b^ | 0.40 (<0.001) | 0.15 (<0.001) | 0.85 (<0.001) | 0.45 (<0.001) | -0.18 (<0.001) | NR | -0.18 (<0.001) |
| CDC N2  (Fecal) | 0.57 (<0.001) | -0.07 (<0.001) | 0.39 (<0.001) | 0.09 (<0.001) | NR | 0.61 (<0.001) | 0.83 (<0.001) | 0.83 (<0.001) | NR | 0.07 (0.286)^a^  -0.03 (0.4)^c^ | -0.65 (<0.001)^c^ | 0.07 (0.286)  -0.06 (0.004)^c^ |
| CDC N1  (Flow & Fecal) | 0.61 (<0.001) | 0.06 (<0.001) | 0.54 (<0.001) | 0.21  (<0.001) | 0.50  (<0.001)^a^  0.60  (<0.001)^b^ | 0.43 (<0.001) | 0.14 (<0.001) | 0.85 (<0.001) | 0.48 (<0.001) | -0.15  (<0.001) | NR | -0.15  (<0.001) |
| CDC N2  (Flow & Fecal) | 0.65 (<0.001) | 0.0002 (0.99) | 0.51 (<0.001) | 0.17 (0.007) | NR | 0.62 (<0.001) | 0.83 (<0.001) | 0.83 (<0.001) | NR | 0.11 (0.093)^a^  -0.06 (0.073)^c^ | -0.52 (<0.001)^c^ | 0.11 (0.093)^a^  -0.09 (0.004)^c^ |

^a^ PMMoV

^b^ F+ Coliphage

^c^ crAssphage
