# Supplementary material for: A multistate assessment of population normalization factors for wastewater-based epidemiology of COVID-19
Source: PLoS One. 2023 Apr 12;18(4):e0284370. doi: 10.1371/journal.pone.0284370 (PMC10096268; doi:10.1371/journal.pone.0284370)
Supplement: Attachment — Submitted filename: Response to Reviewers.docx [file pone.0284370.s004.docx]

**Response to Editor and Reviewers’ Comments**

Our responses are in red font with italics

Editor’s Comments

1. Please ensure that your manuscript meets PLOS ONE's style requirements, including those for file naming.

*Formatting edits have been made throughout the revised manuscript document.*

2. We note that the grant information you provided in the ‘Funding Information’ and ‘Financial Disclosure’ sections do not match. When you resubmit, please ensure that you provide the correct grant numbers for the awards you received for your study in the ‘Funding Information’ section.

*This has been completed.*

3. *In your Data Availability statement, you have not specified where the minimal data set underlying the results described in your manuscript can be found. PLOS defines a study's minimal data set as the underlying data used to reach the conclusions drawn in the manuscript and any additional data required to replicate the reported study findings in their entirety. All PLOS journals require that the minimal data set be made fully available.*

*CDC NWSS data that was used for this study is publicly available and can be found at the following link:* [*https://data.cdc.gov/Public-Health-Surveillance/NWSS-Public-SARS-CoV-2-Wastewater-Metric-Data/2ew6-ywp6*](https://data.cdc.gov/Public-Health-Surveillance/NWSS-Public-SARS-CoV-2-Wastewater-Metric-Data/2ew6-ywp6)

Reviewer #1: The paper face a very important problem for Wastewater Based Epidemiology. The used methods are appropriate and well explained, the literature updated.

1. Did the sewershed population vary along the time, for example due to the summer holidays?

*The reported population for each sewershed did not vary in this dataset and was reported constantly with the same population served value for all data points. This was noted in the track changed manuscript (Lines 257-260).*

2. Are cases reported as resident or present in the studied areas at the time of sampling?

*CDC NWSS provided COVID-19 case data that was primarily collected and uploaded by each U.S. state. According to CDC, this data undergoes an additional quality check procedure to ensure accuracy of COVID-19 reporting (*[*https://www.cdc.gov/coronavirus/2019-ncov/covid-data/faq-surveillance.html*](https://www.cdc.gov/coronavirus/2019-ncov/covid-data/faq-surveillance.html)*). Our assumption throughout this study was that reported cases were for individuals that are residents within each key sewershed and presumptively present in that key sewershed geographic area during the duration of infection.*

3. The length of the sewerage was not taken in account for fecal normalization: could it affect the results increasing the variability of fecal indicators load?

*While sewage travel time is a variable in our CDC NWSS dataset, it was only reported in 11% of the total data set, and only in two of the six study states (California and Ohio). Even among these two states, sewage travel time was not reported for all key sewersheds within each state. Currently, reporting states are not required to provide data for all variables of interest by CDC NWSS prior to upload for public use, which is why the majority of the data set has missing values for travel time. We did consider including sewage travel time when beginning this project. However, due to the above-mentioned limitations with the dataset, we decided not to include this variable in our study analysis.*

2. Reviewer #2: The article titled: A multistate assessment of population normalization factors for wastewater-based epidemiology of COVID-19" is a good, recent and very useful work. I recommend it for publication in your journal in a present form.

*This reviewer had no additional comments.*
